# On Hyperæsthesis Cerebralis

**Published:** 1853-12

**Authors:** T. B. Brown

**Affiliations:** Chicago


*On "Hyperæsthesis Cerebralis:"* By T. B. Brown, M. D.

Among the various forms of diseases, which present themselves to the physician as objects of observation and treatment, those of the central nervous system, together with its membranes, may certainly occupy one of the highest places. It is the especial merit of modern medical science, that the material accumulated about this topic has been successfully arranged, and based upon the results of the pathological anatomy. How great the varieties of these forms of diseases are, every one will confess, who has had any experience whatever. Every practitioner will agree, that in such a vast field there is still a great deal left to be done. Therefore I undertook to describe a disease, which three times I have had the opportunity to observe, and which, in regard to its symptoms and course, may not be uninteresting to many of your readers.

*Symptoms.*—The patients I saw suffering applied for medical assistance on account of being troubled by the most intolerable headache. On questioning them, they all agreed that their sickness had commenced with a certain ill-temper. They had no mind to work, and were struck with sadness, discouragement, anger and apathy for every trifling thing. That stage however lasted but a short time. Suddenly flying and stinging pains were felt through the head, being at first but slight and coming on at longer intervals, but soon increasing to the most intense violence and changing into the most severe headache, interrupted only by a few painless pauses. With the beginning of the now continued and piercing pain, spread over the whole head, a derangement of the general feeling appeared. The patients lost their appetite entirely, a general relaxation coming on, and impossibility of thinking or acting freely. Sleep was disturbed, short, and vexed by dreams. For the rest the temperature of the head as well as that of the trunk was not raised, the skin not turgent, the pulse still normal, or under the greatest pains scarcely perceptibly irritated, the carotid and temporal arteries did not pulsate abnormally. There was no ringing of the ears, neither a complaint of seeing sparks, nor were the eyes glassy, injected, or painful to light. The head was indeed frequently tossed, being full of ideas and wandering; the headache was also increased by stopping the respiration, but there was no nestling the head into the pillow.—The pupils were not dilated, they reacted as in a normal state.—The tongue was not covered, the taste not altered. There were no pains in the extremities or only of a very slight character. The process of respiration was performed without difficulty; no complaint about a pain in the Praecordia. The abdomen was not bloated, the excretions of the alimentary and urinary organs not checked. That stage of the affection of the head was limited to from three to four days, whereupon gradually other appearances took place.

In connection with the headache at this stage, a violent pain in all the extremities came on, which caused the patients to scream frequently. Tossing desperately in the bed, they were unconscious of their position. It is not a tearing, stinging pain, such as we find in rheumatism, when it attacks the greater or smaller joints or other parts of the system; nor is it the peculiar pain of the limbs occurring in many of the gastric or bilious forms, reflected by the spinal system, for this never shows such an intensity, extent and duration. Likewise it is not the characteristic pain of the arthritis (gout), which in paroxysms, exacerbating under the influence of warmth, still increases and only by keeping quiet after a considerable while ceases again. The pain is neither confined to the joints affected with arthritis, nor has it the nature of such a one; but it attacks suddenly all the extremities, and consists of an extremely smarting, piercing, destroying sensation, so that, as the patients express themselves, their limbs make an impression as being nearly crushed, and finally are almost senseless from the severity of the pain.

This suffering is to be compared somewhat with that with which typhus patients are affected sometimes on either or both of the lower extremities, as premonitory symptoms of Scrobutus. In such cases the patients define the pain almost in the same manner. From this moment sleep disappears altogether, and it is with weeping and loud complaints that the unhappy patient implores the physician to relieve him from the continually lasting and excruciating pains of the head and extremities.

This stage pursued its course for from two to three days and still longer, without other groups of symptoms appearing except an augmented temperature of the head. All the secretions and excretions going on regularly, the patient still possessed of consciousness. A change takes place, however, at once. The sensorium, deranged as it was in some manner already, becomes still more deeply affected, for the patient is earnestly complaining that he cannot see well. Everything is vanishing before his eye. The mental disturbance becomes complete. Being asked again he declares he sees different colors before his eyes, the objects appear to him now yellow, then blue, green, red, etc; and then they are getting darker gradually, and at length it looks to him, as if between the eye and object in view, something would pass down obscuring the sight. But all that is learned only with difficulty now from the patient, and it is mostly the result of his later remembrance, which reaches only to that point. Everything following is lost to his memory entirely. He is ignorant where he is, is losing his sense, as he says himself, the faculty of coaptation being almost entirely suspended, From this moment all consciousness is completely lost, and delirium is taking place. This may be looked upon as especially remarkable in its nature, for the reason that the delirium of any other disease of the brain is not to be compared with it, either in regard to intensity or duration. The other symptoms are in no way in proportion to this raging excitement. The most extraordinary tide of ideas is manifested; image after image is produced by the perverse imagination of the patient; the place of one, scarcely half carried through, being filled up by another. Now they are employed with domestic affairs, then they are suddenly deprived of their life in a most dreadful manner. Now they are about to disclose their most profound secrets, but scarcely having commenced, they are talking the wildest nonsense. And so the delirium goes on for three or four days without the least remission in the morning or evening. During that time the head of the patient is moderately warm, being tossed sometimes more sometimes less frequently, but never put into the pillows. The carotids as well as the temporal arteries do not pulsate as in inflammation. The eyes are rapidly opened, glancing indeed, but not injected, nor affected by light. There is no apparent strabismus, nor are they turned to any particular direction in a spasmodic manner, as happens in encephalitis. The pupils are not contracted, nor dilated, and react readily. The countenance shows no appearance of wildness, no threatening expression, it is but moderately reddened, the turgesence being slight.—Gnashing of the teeth I never noticed. The tongue is not trembling, it is clean and often dry. Whether the thirst is violent or not, it is difficult to ascertain. The patient eats and drinks, if anything is brought to his mouth. The pulse may be easier suppressed, is not irregular, but only somewhat accelerated; being from 80 to 90 per minute. The agitation of the hands is slight. The patient but seldom grasps the head, the muscular power of the arms is not augmented. Neither here, nor in the head, nor in other parts of the system do spasmodic contraction of the muscle appear; there is also neither vomiting, nor paralysis, nor flocci-legium, subsultus tendinum, nor any later symptoms of cerebral depression manifested by sopor or coma.

As for the appearance of the breast, the temperature of the skin is not increased, there is no sweating, the action of the heart is somewhat less distinct. The Thorax is raised perfectly, and the process of respiration is going on normally. The abdomen in this stage is, in consequence of the retained fæces, somewhat distended, but still there is no pain in pressing upon it. The urine is scanty and contains an excess of salts. The lower extremities like the upper ones are seldom moved, and they are never contracted by spasms. It may appear remarkable, that with this mental derangement, with this dynamic affection of the medullary substance, even after the loss of the faculty of coaptation, not all the main functions of organic life are disturbed. There is left yet for sustaining life : action of the heart, respiration, pulse, nutrition, etc., but sleep has disappeared. The excretion of urine and fæces has become disturbed.

In the manner already described and under such symptoms the patient continues to be delirious, without the least remission taking place for from six hours to three or four days. Suddenly the delirium ceases consciousness returns. He recognizes his locality, clearly distinguishing every obiect present. The pulse is, in regard to its quantity and quality, just as it was when the delirium commenced. The heat of the head is diminishing, refreshing sleep generally comes on, and in a few hours all the symptoms of his previous condition have entirely passed away without leaving on his memory the least trace of the strange forms which his imagination had devised.

The patient now complains only of considerable pain in the limbs. There is little appetite, the taste not altered. The secretion of urine goes on normally, the fæce s, heretofore retained, are discharged again. Thus the patient continues during a remission of from ten hours to a whole day, without any further change being perceivable.

But now the characteristic lancing pain through the head returns, premonitory of the depressing headache. The patient knows it already, and is mostly weeping in advance on account of the pains, which are not long anticipated. With this beginning, the pulse and warmth of the head are increased again. All the above mentioned symptoms appear gradually, until sight is disturbed and the delirium with all the accessory symptoms commence again, and lasting for thirty or more days, in which the paroxysms present no peculiar features. The body itself after so long a time is not affected at all in proportion to the mental derangement that has been reaching the highest degree of intensity. No apparent debility and emaciation shows itself, no pallor of complexion, etc.; on the contrary, the patients look healthy, a fact indicating that the disease interferes but little if any, with the performance of the functions of assimulation and nutrition.

*Termination.*—This takes place in two different ways. In the first, the delirium scarcely presents any remissions; it lasts longer, and becomes calmer. The pulse is getting smaller, qucicker, thread-like, and it is evident that the patient approaches a state of sopor and coma, accompanied with the well known symptoms as floccilegium, subsultus tendinum, involuntary discharge of the bowels and urinary organs, tension of the abdomen, stertorous respiration, glue-like sweats, covered tongue, and the unhappy patient, who has been afflicted with erethismus before, is dying under the symptoms of oppression and Paralysis of the brain.

In the second place, the delirium occurs at longer intervals, loses gradually its intensity, and is of less duration; the pains of the limbs cease apparently at the same time with the cephalagia; the patient becomes more quiet, and not only remission but also complete apyrexaia takes place, appetite and strength returns, the temperature of the skin becomes normal again, the abdomen is relieved of its tension, all the secretions, as well as excretions, are natural; sleep also becomes more and more regular, and the disease now disappears in a manner as mysterious as was that of its invasion.

The patients describe the peculiar sensation of their convalescence, as if a veil would from day to day fall away from the eyes, so that they not only see every object clearer and more distinctly, but are also enabled to meditate about it with more calmness and clearness.

*Diagnosis.*—The observing physician may, by an exact and particular examination of the groups of symptoms as well as of the course of the disease, avoid confounding it with other acute inflammatory affections of the brain. It might lead me perhaps too far, and it would also reach beyond the limits of my power, if I

should undertake to give a full statement in a correlative manner of all the diagnostic differences of this disease, in comparison with other cerebral affections. Nevertheless, I am convinced, that a strict examination of the pulse, of the temperature of the skin, of the general condition, and of the fever itself, and especially of the peculiarly long duration of the paroxysms, will enable the physician without difficulty to make a correct diagnosis.

*Treatment.*—This in its single indications and modifications, is as a "terra incognita," yet to be discovered. But this fact at least is certain, that abstraction of blood, either general or local, has proved decidedly injurious. Experience has established this point beyond a doubt. Sinapisms, blisters, as well as other derivative remedies, have been entirely useless; they did not diminish the evil in the least. In like manner, narcotics seemed to be hurtful. Either the patients went into a state of sopor after administering opium and its preparations in larger doses, or the pains were increased under the influence of smaller doses. In no way however could the delirium be arrested by it. Purgatives were of no evident use. Warm baths were also injurious. But the application of cold, topical as well as general, in the form of effusions, had the most beneficial effect. The ice-cap applied on the head for a long time lessened the pains very much. Shower baths have also been of great benefit. It may appear strange that the patient in the period of convalesence, which is protracted for a considerable length of time, complains of debility almost incessantly, whilst they present a certain staring glance for a long time.

*Post-mortem Examination.*—If the physician has no knowledge of the disease, confounding it with meningitis or encephalitis, and if he comes to the dissecting table, in order to find his diagnosis confirmed, infallible as he thought it, he will be extremely astonished at once not to find anything in the least degree abnormal. He will find a brain so healthy and sound, that he may well take it for a standard. Not the least vessel congested, nowhere in the substance of the brain a trace of hyperaemia, stasis or inflammation; softening, ulceration, or induration; no atrophy, effusion into the ventricles, or infiltration or change of color of the medullary substance, nor a pseudo-plastic deposit. The membranes in a like healthy condition without the slightest injection or exudation, in short in the whole mass of the brain and its coverings there is even with the greatest precaution not the least anatomical lesion to be detected. In regard to the condition of the other organs, the agony may be the real cause of their state, as, for instance of the sanguinolent stasis, or of the œdematous infiltration into the parenchyma of the lungs, in consequence of the progressing paralysis of the nervous vagus etc.

Of those three cases thot I happened to see myself, but one recovered, and that under the influence of a merely expectant treatment, an additional proof that there was no material derangement of the brain. This fact as well as the results of dissection might lead us to the conclusion that the disease must be referred to a dynamic disturbance of the functions of the brain, from a certain immaterial, morbid alteration of the medullary substance. The derangement of the organic function in the commencement of the affection may therefore be explained in a like manner, as at the end of it is the inevitable and rapidly following death, owing to a want of regulation of the innervation and of the vegetative functions in general, from whence also the use of the term "hyperæsthesis cerebralis."

*Aetiology.*—According to the few observations of my own as well as from what I have learned from others, this disease attacks mostly women and the dynamic irritation is produced by too intense emotions as grief from any cause, as death, unfortunate amours, &c.

*Prognosis.*—This is extremely unfavorable, of the cases I have seen or heard of only a very few have recovered.

*Remarks.*—I have thus in brief presented the outlines, very imperfectly I confess, from the fact that my observation has been limited, of a disease which so far as I am aware has neve rbeen described by anyMedical writer either of Europe or this country, and only alluded to by some of the older writers under the term "Mania acuta or Mania acutissima."

More than ten years since my respected teacher Professor Yietl, director of the general hospital at Munich, had his attention called to this disease ; since then he has had six more cases, and at that time suggested the name *Hyperœsthesis cerebralis.* Although this may not perfectly express the nature of the affection, I know of none more applicable. "Encephalitis Nervosa" is objectionable from the fact that it conveys a positively wrong idea. I may say this much in reference to my description of this disease that it is derived entirely from clinical observation. IfI have rendered any service to the science of medicine by what I have written I shall be amply rewarded.

Chicago, Nov., 1853.

